# An mTOR‐Tfeb‐Fabp7a Axis Ameliorates *bag3* Cardiomyopathy via Decelerating Cardiac Aging

**DOI:** 10.1111/acel.70216

**Published:** 2025-09-08

**Authors:** Yonghe Ding, Xueling Ma, Feixiang Yan, Baul Yoon, Wei Wei, Yuji Zhang, Xueying Lin, Xiaolei Xu

**Affiliations:** ^1^ Department of Biochemistry and Molecular Biology, Department of Cardiovascular Medicine Mayo Clinic Rochester Minnesota USA; ^2^ School of Nursing Beijing University of Chinese Medicine Beijing China; ^3^ Department of Epidemiology and Public Health University of Maryland School of Medicine Baltimore Maryland USA

## Abstract

While *BAG3* has been identified as a causative gene for dilated cardiomyopathy, the major pathological events in *BAG3‐*related cardiomyopathy that could be targeted for therapeutic benefit remain to be discovered. Here, we aim to uncover novel pathological events through genetic studies in a zebrafish *bag3* cardiomyopathy model. Given the known cardioprotective effects of *mtor* inhibition and the fact that *transcription factor EB (tfeb*) encodes a direct downstream phosphorylation target of mTOR signaling, we generated a cardiomyocyte‐specific transgenic line overexpressing *tfeb* (*Tg[cmlc2:tfeb]*). This overexpression was sufficient to restore defective proteostasis and rescue cardiac dysfunction in the *bag3* cardiomyopathy model. Importantly, we detected accelerated cardiac senescence in the *bag3* cardiomyopathy model, which can be mitigated by *Tg(cmlc2:tfeb)*. We compared cardiac transcriptomes between the *Tg(cmlc2:tfeb)* transgenic fish and the *mtor* ^*xu015/+*^ mutant and found that inhibition of the fatty acid binding protein a (*fabp7a*) gene exerts therapeutic effects. Consistent with this genetic evidence, we detected elevated *fabp7a* expression in the *bag3* cardiomyopathy model, whereas cardiomyocyte‐specific overexpression of *fabp7a* induced dysregulated proteostasis, accelerated cardiac senescence, and cardiac dysfunction. To elucidate the functions of Fabp7a in normative cardiac aging, we turned to the African Turquoise Killifish. We noted elevated Fabp7a expression in the hearts of aged killifish, and pharmacological inhibition of Fabp7a mitigated the cardiac aging process. Together, this study uncovered accelerated cardiac senescence as a key pathological event in *bag3* cardiomyopathy and reveals that manipulating the mTOR‐Tfeb‐Fabp7a axis can mitigate this pathology and confer cardioprotective effects.

## Graphical abstract

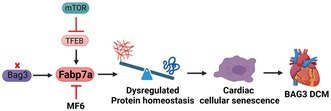

## 1 Introduction

*BAG3* has been recently identified as a causative gene for dilated cardiomyopathies (DCM), which is characterized by ventricular chamber enlargement and systolic dysfunction (Norton et al. 2011; Villard et al. 2011; Mizushima and Sadoshima 2017).

Dysregulated proteostasis, such as altered proteasome or autophagy activity, an imbalance between protein folding and degradation, and the accumulation of misfolded or aggregated proteins in cardiomyocytes, has been identified as a pathological feature of *BAG3*‐associated DCM (McLendon and Robbins 2015). *BAG3* encodes a co‐chaperone protein that binds to heat shock protein 70 and plays a direct and important role in proteostasis, especially in the turnover of sarcomeric proteins (Meriin et al. 2018). Consistent with the notion that dysregulated proteostasis is a key pathological event, our recent study of a *bag3* ^*e2/e2*^ cardiomyopathy model in zebrafish indicated that inhibition of the mechanistic target of rapamycin (mTOR) via an *mtor* haploinsufficiency mutant (*mtor* ^*xu015/+*^) exerted cardioprotective effects by restoring proteostasis (Ding et al. 2019). As a direct downstream phosphorylation target of mTOR signaling (through phosphorylation at S211 in the human TFEB protein) (Napolitano et al. 2018), mammalian TFEB (Transcription factor EB) is a master transcriptional regulator of lysosomal biogenesis and autophagy genes and has been implicated in a diverse range of functions such as nutrient sensing, lipid catabolism, and protein homeostasis (Martina et al. 2012; Tan et al. 2022). Overexpression of *TFEB* has been shown to exert therapeutic benefits for neurodegenerative diseases (Takla et al. 2023) and to repair CryABR^120G^ overexpression‐induced cardiac proteinopathy (Pan et al. 2017). Therefore, *tfeb* is a promising downstream candidate gene that could recapitulate the cardioprotective effects of mTOR inhibition on the *bag3* ^*e2/e2*^ cardiomyopathy model via repairing dysregulated proteostasis. However, experimental evidence is required to test this hypothesis.

Aging is a significant risk factor for cardiac diseases (North and Sinclair 2012; Rodgers et al. 2019), and dysregulated proteostasis has been proposed as one of the 12 hallmarks of normative aging (Lopez‐Otin et al. 2023). Besides dysregulated proteostasis, accelerated cardiac senescence is another hallmark of cardiac aging that has been reported to contribute to the pathogenesis of certain types of cardiomyopathies such as anthracycline‐induced cardiotoxicity (AIC) (Linders et al. 2024). While the expression of Bag3 protein has been linked to aging, as evidenced by a significantly elevated Bag3/Bag1 ratio in the neurons of aged rodent brain (Gamerdinger et al. 2009), it remains unclear whether cardiac senescence contributes to the pathogenesis of *bag3* ^*e2/e2*^ cardiomyopathy.

Fatty acid binding proteins (FABP) are a family of intracellular lipid‐binding proteins mostly known for acting as lipid chaperones that mediate lipid transport and maintain lipid metabolic homeostasis. In humans, at least 10 FABPs have been identified and categorized based on their expression levels in various tissues and organs (Smathers and Petersen 2011). Several FABP family members, such as FABP3, FABP4, and FABP9, have been identified as potential downstream target genes of TFEB, playing roles in cellular lipid metabolic process (Palmieri et al. 2011). FABP3, also known as the heart‐specific FABP, is most abundantly expressed in the heart and cardiac muscle, emerging as a promising biomarker for coronary and peripheral artery disease (Rezar et al. 2020). In contrast, FABP7, referred to as the brain‐type FABP, mainly functions in brain development, learning and memory, sleeping disorders, and tumorigenesis (George Warren et al. 2024; Gerstner et al. 2017). To date, there are no reports on the expression or functions of Fabp7 in the heart or in cardiac diseases.

Here, we aim to leverage the efficient genetic tools offered by zebrafish to elucidate the pathogenesis of *bag3* ^*e2/e2*^ cardiomyopathy and explore novel therapeutic avenues. First, we characterized a transgenic line with cardiomyocyte‐specific overexpression of *tfeb*, and provided experimental evidence to confirm its therapeutic benefits, which recapitulate the cardioprotective effects of *mtor* inhibition. Next, we searched for additional modifying genes by testing differentially expressed genes (DEGs) identified through a transcriptome comparison between the *mtor* inhibition and the *tfeb* transgenic overexpression fish lines. Using an F0‐based genetic screen, we identified *fabp7a* as a new genetic modifier. While the inhibition of fabp7a recapitulated the cardioprotective effects of *mtor* inhibition and *tfeb* overexpression on the *bag3* cardiomyopathy, activation of *fabp7a* exacerbated these phenotypes. Importantly, we noted accelerated cardiac aging in the *bag3* model, which could be mitigated via modulating the mTor‐TFEB‐Fabp7 axis. To provide direct evidence to support the function of Fabp7 in cardiac aging, we utilized the turquoise killifish, known for its short lifespan of (Ruparelia et al. 2024). Our results showed that pharmacological inhibition of Fabp7a via MF6, a specific inhibitor of Fabp7, decelerates normative cardiac aging.

## 2 Results

### 2.1 Cardiomyocyte‐Specific Overexpression of *tfeb* Recapitulated the Cardioprotective Effects of *mtor^xu015/+^* in the *bag3* Cardiomyopathy Model

**FIGURE 1:**
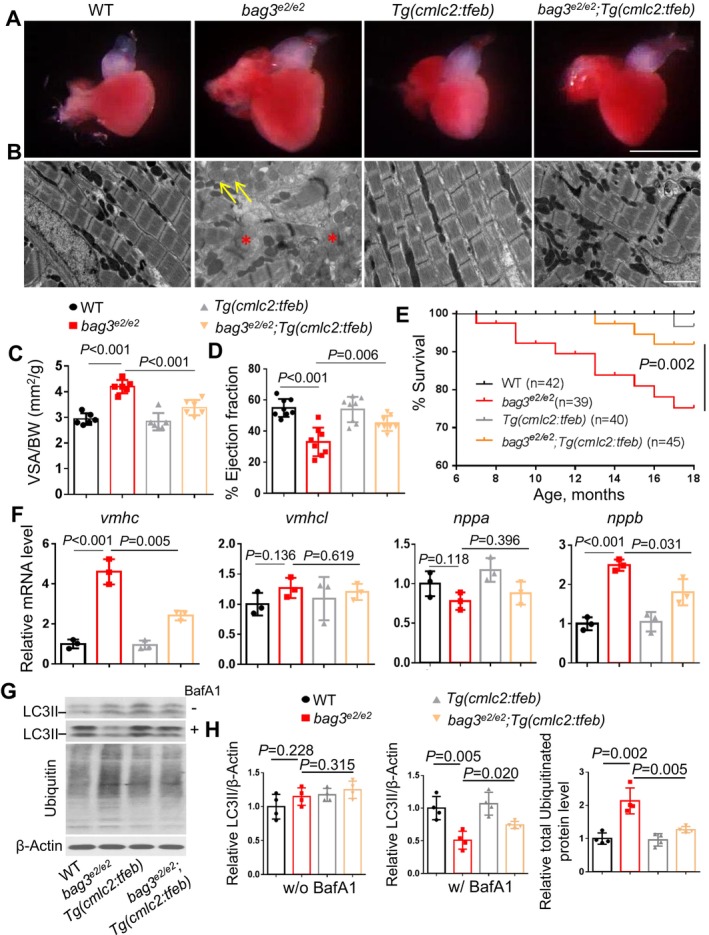
Cardiomyocyte‐specific overexpression of tfeb alleviated bag3 cardiomyopathy phenotypes and restored impaired proteostasis. (A–C) Bright‐field images of dissected hearts (A) confirmative images of transmission electron microscope (TEM) (B) and quantification analysis (C) show enlarged ventricular surface area (VSA) normalized to body weight (BW), impaired sarcomere structure (Asterisks), and mitochondrial swelling (Arrows) phenotypes in the *bag3* ^*e2/e2*^ mutant hearts, which were ameliorated by *Tg(cmlc2:tfeb)*, a cardiomyocyte‐specific *tfeb* overexpression transgenic line. Scale bars in A, 1 mm; in B, 20 μm. (D, E) Ejection fraction (EF) (D) and Survival (in %) (E) of *bag3* ^*e2/e2*^; *Tg(cmlc2:tfeb)* double mutant/transgenic fish compared to the *bag3* ^*e2/e2*^ single mutant, *Tg(cmlc2:tfeb)* single transgenic or WT control fish; *n* = 39–45, log‐rank test. (F) Quantitative RT‐PCR analysis of cardiomyopathy molecular markers in *bag3* ^*e2/e2*^; *Tg(cmlc2:tfeb)* double mutant/transgenic fish compared to single‐mutant/transgenic fish and WT control fish. *n* = 3 biological replicates, one‐way ANOVA. (G–H) Western blot (G) and quantification analysis of the LC3 II and ubiquitinated proteins in indicated fish heart treated with or without 50 nM bafilomycin A1 (BafA1) for 4 h. *n* = 4 biological replicates, one‐way ANOVA.

We recently reported that a frameshift mutation in the second exon of the zebrafish *bag3* gene resulted in cardiomyopathy‐like phenotypes at 6 months of age, termed *bag3* ^*e2/e2*^ cardiomyopathy (Ding et al. 2019). A zebrafish mechanistic target of rapamycin (mTOR) haploinsufficiency mutant (*mtor* ^*xu015/+*^) exerted cardioprotective effects. Because Tfeb is a direct downstream phosphorylation target of mTOR signaling, we investigated whether the cardioprotective effects of mTOR inhibition can be recapitulated by Tfeb overexpression. We tested *Tg(cmlc2:tfeb)*, a transgenic fish line with cardiomyocyte‐specific overexpression of *tfeb* (Kim et al. 2021), and found that the *Tg(cmlc2:tfeb)* transgene effectively rescued cardiomyopathy‐like phenotypes including increased ventricular chamber size, damaged myocardium structure, reduced cardiac function, shortened lifespan, and re‐activation of fetal gene programs in the *bag3* ^*e2/e2*^ mutant fish (Figure 1A–F). Because dysregulated proteostasis is a major pathological feature of *bag3* cardiomyopathy, and dysregulation of autophagy, a dynamic process involving both the formation and clearance of autophagosomes, often contributes to proteostasis disruption (McLendon and Robbins 2015), we evaluated autophagy in *bag3* ^*e2/e2*^ mutant fish hearts with or without the *Tg(cmlc2:tfeb)* transgene. Our results revealed significantly impaired autophagic flux in *bag3* ^*e2/e2*^ mutant fish hearts, as indicated by the failure of LC3 II to accumulate in response to the autophagy inhibitor bafilomycin A1 (BafA1) treatment and by elevated total ubiquitinated protein levels. Importantly, these defects were partially restored by the *Tg(cmlc2:tfeb)* transgene (Figure 1G,H). Collectively, these data suggest that cardiomyocyte‐specific overexpression of *tfeb* recapitulated the therapeutic effects of mTOR inhibition in the *bag3* cardiomyopathy model.

### 2.2 Cardiomyocyte‐Specific Overexpression of *tfeb* Decelerated Cardiac Aging Phenotypes in the *bag3* Cardiomyopathy Model

**FIGURE 2:**
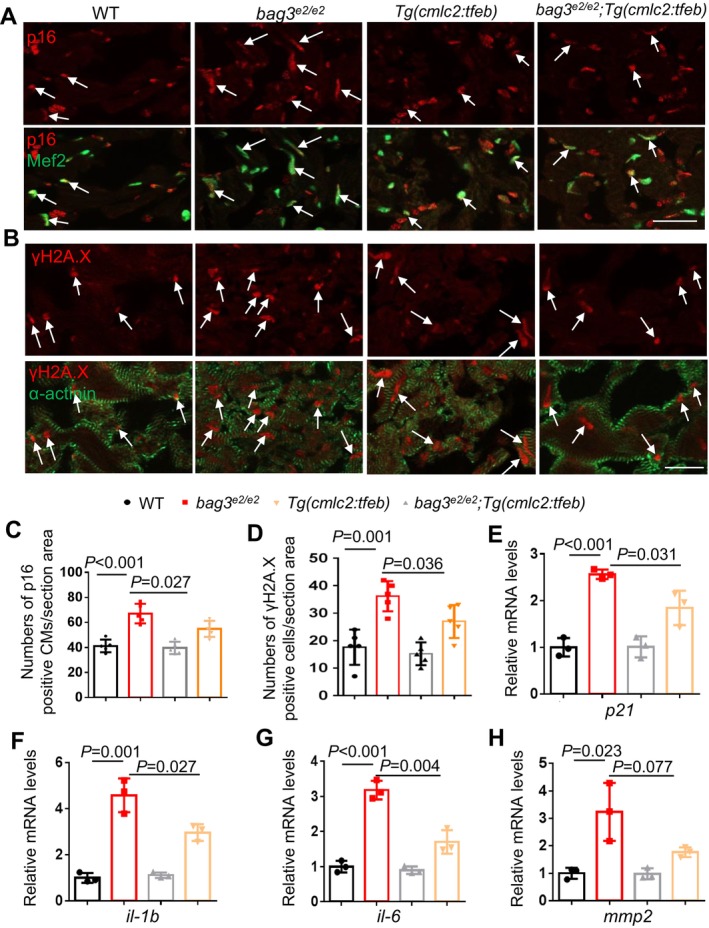
Cardiomyocyte‐specific overexpression of *tfeb* decelerated cardiomyocyte senescence. (A, B) Representative images of cryosectioned heart tissues co‐immunostained using either anti‐p16 antibody co‐stained with anti‐Mef2 antibody (A) or anti‐γH2A antibody co‐stained with anti‐α‐actinin antibody (B) in the *bag3* ^*e2/e2*^; *Tg(cmlc2:tfeb)* double‐mutant/transgenic fish compared to single‐mutant/transgenic fish and WT control fish at 6 months. Scale bars: 20 μm. Arrows point to overlapping signals. (C, D) Quantification of the numbers of p16/Mef2 and γH2A.X/α‐actinin antibodies co‐immunostained cells shown in A and B. *n* = 5, One‐way ANOVA. (E–H) Quantitative RT‐PCR analysis of cellular senescence marker p21 and senescence‐associated secretory phenotype (*SASP*) markers in *bag3* ^*e2/e2*^; *Tg(cmlc2:tfeb)* double mutant/transgenic fish compared to single‐mutant/transgenic fish and WT control fish. *n* = 3 biological replicates, one‐way ANOVA.

Upon closer examination of the *bag3* ^*e2/e2*^ mutants, we observed a curved body shape in some of *bag3* ^*e2/e2*^ mutants at 6 months of age (Figure S1A), prompting us to investigate aging‐related phenotypes. We detected increased senescence‐associated (SA) β‐galactosidase activity throughout the body and in the heart at 6 months of age, with more prominent signals in the atrium than in the ventricle (Figure S1B). However, the overall β‐gal staining signal in the adult zebrafish heart chambers, especially in the ventricle, was very faint, with only a few dots detected. We next performed immunofluorescent staining of sectioned hearts and observed a significantly increased signal of the cellular senescence marker p16 and DNA damage marker γH2A.X in the cardiomyocytes of *bag3* ^*e2/e2*^ mutant fish hearts at 6 months of age (Figure 2A–D). In addition, we carried out quantitative RT‐PCR experiments and found significantly elevated transcripts of *p21*, a well‐recognized cellular senescence marker in zebrafish (Morsli et al. 2023), and senescence‐associated secretory phenotype (*SASP*) markers including *il‐1b*, *il‐6*, and *mmp2* in the *bag3* ^*e2/e2*^ mutant fish hearts (Morsli et al. 2023; Vanhunsel et al. 2021) (Figure 2E–H, Figure S1C–F). Importantly, these aging‐associated phenotypic markers were attenuated by the *Tg(cmlc2:tfeb)* transgene (Figure 2). Together, these results strongly suggest that accelerated cardiac senescence is a pathological event in the *bag3* cardiomyopathy model, which can be ameliorated by cardiomyocyte‐specific *tfeb* activation.

### 2.3 Genetic Analysis of Shared Differentially Expressed Genes (DEGs) Between *Tg*(*cmlc2:tfeb*) and *mtor^xu015/+^* Identified *fabp7a* as a Therapeutic Modifier Gene for *bag3* Cardiomyopathy

**FIGURE 3:**
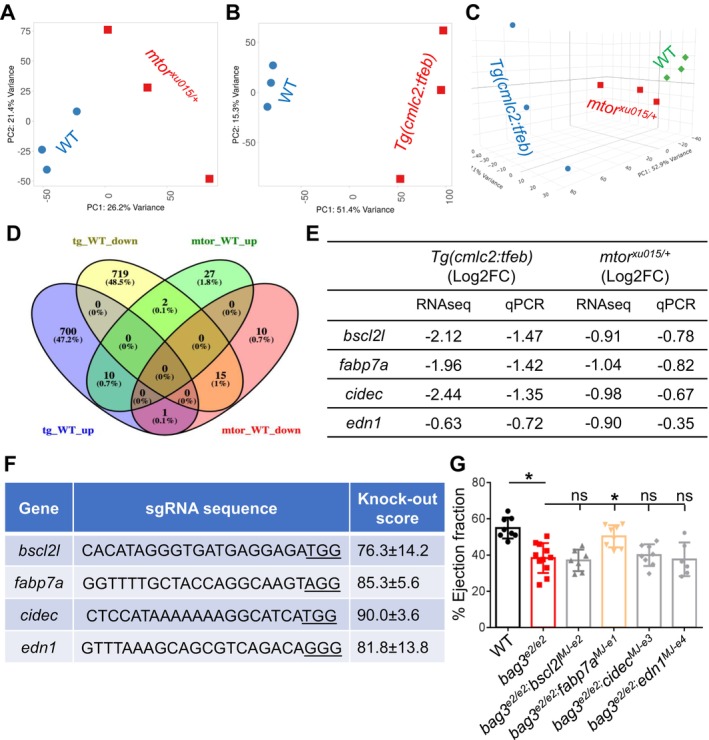
Combination of RNA‐seq with an F0‐based genetic screen identified *fabp7a* as a therapeutic modifier gene for the *bag3* cardiomyopathy model. (A, B) Principal Component Analysis (PCA) of RNA‐seq‐based expression data from the *mtor* ^*xu015/+*^ mutant (A) or *Tg(cmlc2:tfeb)* (B) transgenic fish hearts compared to WT controls. (C) 3D PCA plot using the normalized gene expression of all expressed genes to assess the genome‐wide transcriptomic similarity among *mtor* ^*xu015/+*^ mutant, *Tg(cmlc2:tfeb)* transgenic fish hearts, and WT controls. (D) Comparison of cardiac transcriptome between *mtor* ^*xu015/+*^ mutant and *Tg(cmlc2:tfeb)* transgenic fish hearts identified 10 upregulated and 15 downregulated genes as common DEGs. (E) Quantitative RT‐PCR validated the expression of 4 lipodystrophic DE genes that were downregulated in both the *mtor* ^*xu015/+*^ mutant and the *Tg(cmlc2:tfeb)* transgenic hearts. (F) List of single guide RNA (sgRNA) sequences for the 4 lipodystrophic DE genes and knockout scores detected from the adult fish injected with sgRNAs in F0 generation. (G) Injection of *fabp7a* microhomology‐mediated end joining (MMEJ)‐inducing sgRNA, but not the other 3 individual MMEJ sgRNAs, exerts a cardioprotective effect on *bag3* ^*e2/e2*^ cardiomyopathy in F0 adult fish.

To explore the molecular mechanisms underlying the cardioprotective effects of *mtor* ^*xu015/+*^ haploinsufficiency mutants and *Tg(cmlc2:tfeb)* transgenes on *bag3* cardiomyopathy, we compared their transcriptomes through RNA‐sequencing analysis of whole heart tissues. Principal component analysis (PCA) revealed that the cardiac transcriptomes of either *mtor* ^*xu015/+*^ haploinsufficiency mutants or *Tg(cmlc2:tfeb)* transgenes formed clusters distinct from the corresponding WT sibling control samples (Figure 3A,B). Using a cut‐off of an adjusted *p*‐value < 0.05 and ≥ 1.5‐fold changes, we identified 1936 differentially expressed genes (DEG) in the *Tg(cmlc2:tfeb)* versus WT, and 65 DEGs in the *mtor* ^*xu015/+*^ versus WT (Figure S2). Notably, we found that the *mtor* ^*xu015/+*^ samples clustered more closely with WT than the *Tg(cmlc2:tfeb)* samples when using normalized gene expression of all expressed genes to assess genome‐wide transcriptomic similarity among these three groups (Figure 3C). Given that TFEB functions downstream of mTOR, its overexpression can likely bypass upstream mTOR regulation and drive broader transcriptional reprogramming, whereas partial mTOR haploinsufficiency in the *mtor* ^*xu015/+*^ mutant induces more limited transcriptomic changes, likely buffered by compensatory pathways.

The top pathways in the *mtor* ^*xu015/+*^ haploinsufficiency mutants include circadian clock, protein folding, and macrophage alternative activation signaling pathway (Figure S2). In contrast, the top pathways in the *Tg(cmlc2:tfeb)* transgene lines include cell cycle checkpoints, mitotic prometaphase, and activation of the pre‐replicative complex. We identified 25 overlapping genes between these two DEG populations, with 15 downregulated and 10 upregulated (Figure 3D). Intriguingly, among the 15 overlapping downregulated genes, we noted 4 lipodystrophy genes: *bscl2l, fabp7a, cidec*, and *edn1* (Figure 3E, Table S1). We chose to test potential modifying effects of these 4 lipodystrophy genes on the *bag3* cardiomyopathy model using an F0‐based genetic assay (Ding et al. 2023). We designed microhomology‐mediated end joining (MMEJ)‐inducing single guide RNAs (sgRNAs) and tested their knockout efficiency via injecting into wildtype embryos. After confirming high knockout efficiency, that is, ranging from 76% to 90% (Figure 3F), we injected each of the 4 sgRNAs into the offsprings of *bag3* ^*e2/+*^ incrosses. We found that only the sgRNA targeting the first exon of the *fabp7a* gene (*fabp7a* ^*MJ‐e1*^), but not the other 3 lipodystrophy genes such as *bscl2l, cidec*, or *edn1*, restored the cardiac dysfunction in the *bag3* cardiomyopathy model at 6 months of age (Figure 3G). This data suggested that *bscl2l, cidec*, and *edn1* genes are likely involved in different pathways from *fabp7a*. Nevertheless, our genetic studies suggested that *fabp7a* is a potential therapeutic modifier gene for *bag3* cardiomyopathy.

**FIGURE 4:**
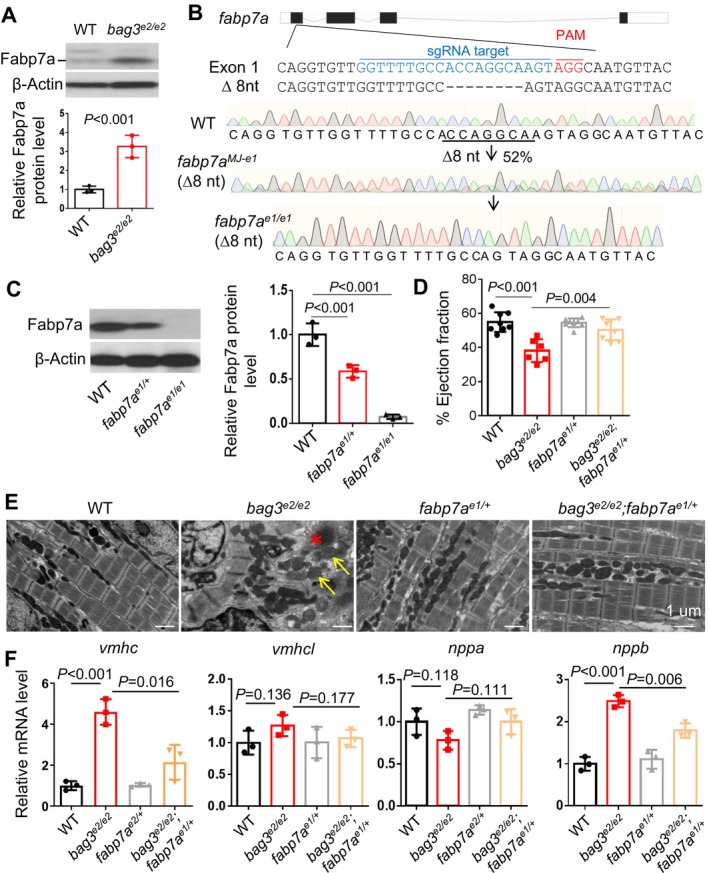
The therapeutic modifying effects of *fabp7a* inhibition were validated in the stable F1 haploinsufficiency mutant. (A) Western blot and quantification analysis of the Fabp7a protein levels in heart lysates of *bag3* ^*e2/e2*^ mutant compared to WT control fish hearts at 6 months. (B) Schematic and chromatographs illustrating the genetic lesion of 8 nucleotides generated by injection of an MMEJ inducing *fabp7a* sgRNA targeting sequences within the first exon. Dashed lines indicate an 8‐nucleotide‐long deletion. (C) Western blot and quantification analysis of the Fabp7a protein levels in the *fabp7a* stable heterozygous (*fabp7a* ^*e1/+*^) and homozygous (*fabp7a* ^*e1/e1*^) mutant fish compared to WT controls. *n* = 3, one‐way ANOVA. (D) Quantification of cardiac function. Ejection fraction (EF) (in %) measured by echocardiography in *bag3* ^*e2/e2*^; *fabp7a* ^*e1/+*^ double mutant fish compared to single mutant and WT control fish at 6 months; *n* = 6–8, one‐way ANOVA. (E) Confirmative TEM images of *bag3* ^*e2/e2*^; *fabp7a* ^*e1/+*^ double mutant fish hearts compared to *bag3* ^*e2/e2*^ and *fabp7a* ^*e1/+*^ single mutant and WT controls at 6 months. The asterisk indicates Z‐disc aggregation. Arrows point to mitochondrial rounding and swelling. Scale bar: 2 μm. (F) Quantitative RT‐PCR analysis of cardiomyopathy molecular markers in *bag3* ^*e2/e2*^; *fabp7a* ^*e1/+*^ double mutant fish compared to single mutant and WT control fish at 6 months. *n* = 3 biological replicates, one‐way ANOVA.

Next, we performed a Western blot analysis and detected significantly elevated expression of the Fabp7a protein in the *bag3* cardiomyopathy model compared to WT control fish hearts (Figure 4A). To confirm the salutary modifying effect of *fabp7a* inhibition on *bag3* cardiomyopathy, we generated a stable *fabp7a* mutant that harbors an 8‐nucleotide deletion (Figure 4B). At the protein level, the expression level of Fabp7a was reduced to 57% in the *fabp7a* ^*e1/+*^ heterozygous mutant and 3% in the *fabp7a* ^*e1/e1*^ homozygous mutant fish compared to that in WT control (Figure 4C). We found that cardiac dysfunction in the *bag3* ^*e2/e2*^ mutant fish at 6 months of age could be partially rescued in the *bag3* ^*e2/e2*^; *fabp7a* ^*e1/+*^ double‐mutant fish (Figure 4D). Both sarcomeric damage and mitochondrial swelling phenotypes were largely restored in the *bag3* ^*e2/e2*^; *fabp7a* ^*e1/+*^ double‐mutant fish (Figure 4E). At the molecular level, re‐activation of fetal gene programs detected in the *bag3* ^*e2/e2*^ mutant hearts was partially inhibited in the *bag3* ^*e2/e2*^; *fabp7a* ^*e1/+*^ double‐mutant hearts (Figure 4F). Collectively, these data confirm *fabp7a* as a modifier gene for *bag3* cardiomyopathy that can be inhibited to exert cardioprotective effects.

### 2.4 *fabp7a* Inhibition Restored Dysregulated Proteostasis and Decelerated Cardiac Aging Phenotypes in the *bag3* Cardiomyopathy Model

**FIGURE 5:**
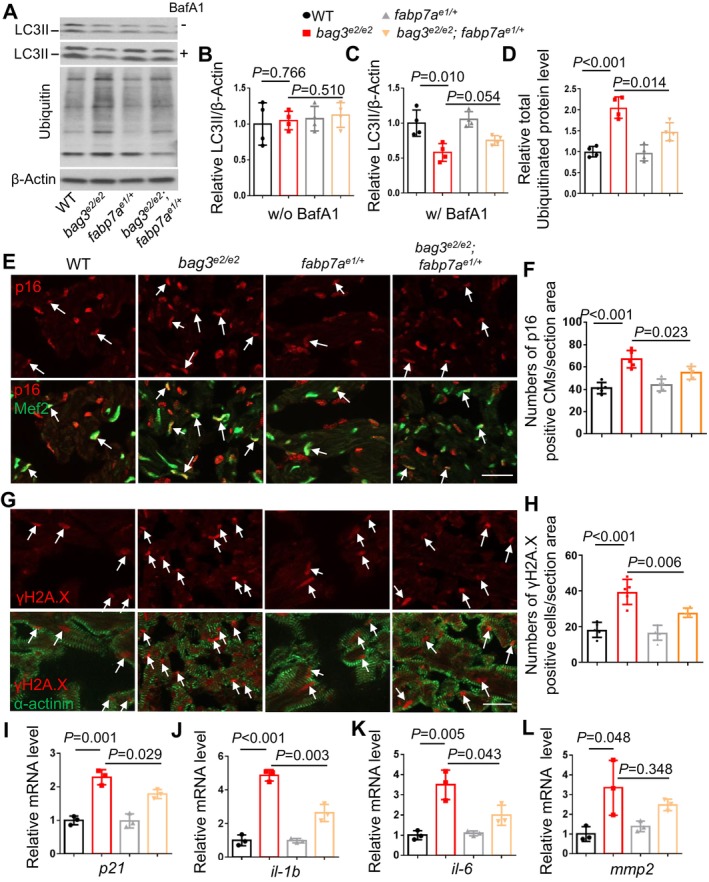
*fabp7a* inhibition is sufficient to restore impaired protein homeostasis and decelerate cardiomyocyte senescence in the *bag3* cardiomyopathy model. (A–D) Western blot (A) and quantification analysis of LC3II with (B) or without (C) BafA1 treatment, and ubiquitinated protein (D) levels in heart lysates of *bag3* ^*e2/e2*^; *fabp7a* ^*e1/+*^ double‐mutant, *bag3* ^*e2/e2*^, *fabp7a* ^*e1/+*^ single mutant, or WT control fish hearts at 6 months treated with or without 50 nM BafA1 for 4 h. *n* = 4, one‐way ANOVA. (E, F) Representative images of immunostaining (E) and quantification of the numbers of p16/Mef2 signal (F) in cryosectioned heart tissues co‐stained with anti‐p16 and anti‐Mef2 antibodies. G‐H, Representative images of immunostaining (G) and quantification of the numbers of γH2A.X/α‐actinin signal (H) in cryosectioned heart tissues co‐stained with γH2A.X and anti‐α‐actinin antibodies (G) in *bag3* ^*e2/e2*^; *fabp7a* ^*e1/+*^ double‐mutant, *bag3* ^*e2/e2*^, *fabp7a* ^*e1/+*^ single mutant, or WT control fish hearts at 6 months. Arrows point to overlapping signals. Scale bars: 20 μm. *n* = 5, one‐way ANOVA. (I–L) Quantitative RT‐PCR analysis of cellular senescence marker p21 and *SASP* markers in *bag3* ^*e2/e2*^; *fabp7a* ^*e1/+*^ double‐mutant compared to single‐mutant fish and WT control fish hearts at 6 months. *n* = 3 biological replicates, one‐way ANOVA.

Next, we investigated whether *fabp7*a inhibition could alleviate proteostasis dysregulation and aberrant autophagic flux. We found that the suppressed autophagic flux in the *bag3* cardiomyopathy model was partially restored by the *fabp7a* ^*e1/+*^ haploinsufficiency mutation, as indicated by our Western blot analysis results of LC3 II levels with or without BafA1 treatment (Figure 5A–C). We also found significantly increased expression levels of the chaperone protein Hsp70, but not Hsc70, as well as elevated levels of both total and aggregated ubiquitinated proteins in the *bag3* cardiomyopathy model that were partially rescued by the *fabp7a* ^*e1/+*^ haploinsufficiency mutation (Figure S3). In addition, the increased apoptosis index while reduced proteasome activity in the *bag3* cardiomyopathy model were partially rescued by the *fabp7a* ^*e1/+*^ haploinsufficiency mutation (Figure S4). Given that the *bag3* cardiomyopathy model exhibited accelerated cardiac aging phenotypes, we then asked whether *fabp7*a inhibition affects cardiomyocyte senescence through immunostaining assays. We found that the elevated expression of the cellular senescence marker p16 and DNA damage marker γH2A.X in the *bag3* ^*e2/e2*^ mutant hearts were significantly ameliorated in the *bag3* ^*e2/e2*^; *fabp7a* ^*e1/+*^ double‐mutant hearts (Figure 5C–E). In addition, at the transcript level, the elevated expression of the cellular senescence marker *p21* and *SASP genes* including *il‐1b* and *il‐6*, but not *mmp2*, in the *bag3* ^*e2/e2*^ mutant hearts were also partially inhibited in the *bag3* ^*e2/e2*^; *fabp7a* ^*e1/+*^ double mutant hearts (Figure 5F–I). Together, these results suggest that *fabp7a* inhibition can repair dysregulated proteostasis and rejuvenate cardiomyocytes in the *bag3* cardiomyopathy model.

### 2.5 Cardiomyocyte‐Specific Overexpression of *fabp7a* Caused Cardiomyopathy Accompanied by Dysregulated Proteostasis and Accelerated Cardiac Aging

**FIGURE 6:**
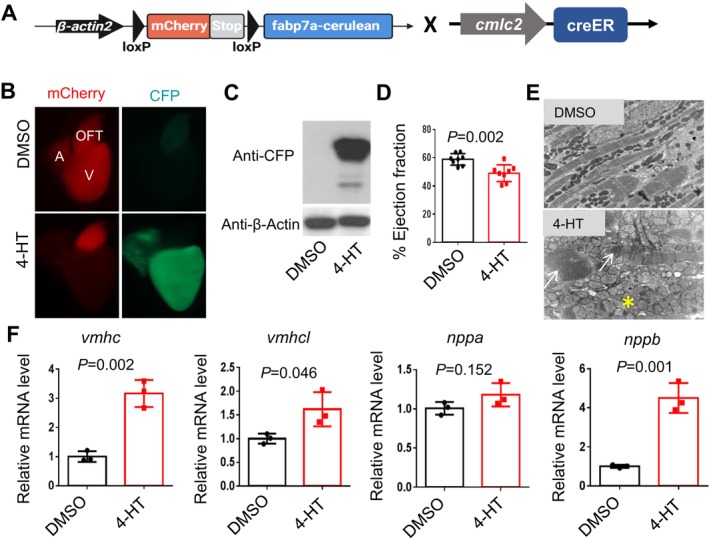
Cardiomyocyte‐specific overexpression of *fabp7a* gene results in cardiac dysfunction. (A) Schematic constructs and design to generate a transgenic line with cardiomyocyte‐specific overexpression of *fabp7a* in zebrafish. (B, C) Cardiomyocyte‐specific overexpression of Fabp7a was indicated by fluorescence switch from mCherry to cerulean fluorescent protein (CFP) (B) and by Western blot analysis (C) at one week post‐4‐Hydroxytamoxifen (4‐HT) induction. (D, E) Cardiomyocyte‐specific overexpression of Fabp7a led to cardiac function decline (D) impaired sarcomere (arrows) and swollen mitochondria (asterisk) (E) at 3 months post‐4‐HT induction. (F) Quantitative RT‐PCR analysis of cardiomyopathy molecular markers in the cardiomyocyte‐specific overexpression of Fabp7a fish at 3 months post‐4‐HT induction. *n* = 3 biological replicates, one‐way ANOVA.

**FIGURE 7:**
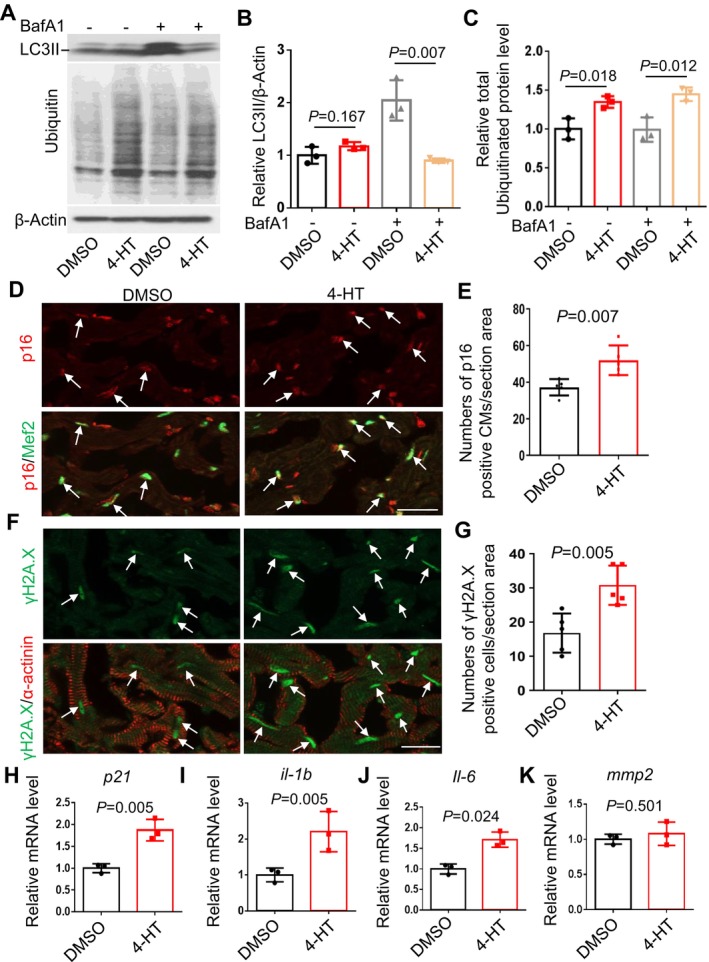
Cardiomyocyte‐specific overexpression of *fabp7a* gene caused impaired protein homeostasis and accelerated cellular senescence. (A–C) Western blot (A) and quantification analysis of the LC3 II (B) and ubiquitinated proteins (B) levels indicated fish heart treated with or without 50 nM BafA1 for 4 h. *n* = 3 biological replicates, one‐way ANOVA. (D, E) Representative images of immunofluorescence using anti‐p16 antibody co‐stained with anti‐Mef2 antibody (D) and quantification of the numbers of anti‐p16/anti‐Mef2 signal (E) in the cardiomyocyte‐specific overexpression of Fabp7a fish at 3 months post‐4‐HT induction. Arrows point to overlapping signals. Scale bars: 20 μm. *n* = 5, one‐way ANOVA. (F, G) Representative images of immunofluorescence using anti‐γH2A.X antibody co‐stained with anti‐α‐actinin antibody (F) and quantification of the numbers of anti‐γH2A.X/anti‐α‐actinin co‐stained cell signal (G) in the cardiomyocyte‐specific overexpression of Fabp7a fish at 3 months post‐4‐HT induction. Scale bars: 20 μm. *n* = 5, one‐way ANOVA. (H–K) Quantitative RT‐PCR analysis of cellular senescence marker *p21* and *SASP* markers including *il‐1b, il‐6*, and *mmp2* in the cardiomyocyte‐specific overexpression of Fabp7a fish at 3 months post‐4‐HT induction. *n* = 3 biological replicates, one‐way ANOVA.

To further elucidate the functions of *fabp7a* in cardiomyopathy, we generated a conditional overexpression line named *Tg(β‐actin2:loxP‐mCherry‐loxP‐fabp7a‐cerulean)*, or *Tg(fabp7a‐OE)* for simplicity in adult zebrafish (Figure 6A). After crossing the *Tg(fabp7a‐OE)* fish with the *Tg(cmlc2‐creERT2)* line that drives cardiomyocyte‐specific Cre expression upon 4‐Hydroxytamoxifen (4‐HT) induction (Kikuchi et al. 2010), we observed an obvious fluorescence switch from mCherry to cerulean in the hearts of the *Tg(fabp7a‐OE*); *Tg(cmlc2‐creER)* double transgenic fish at one week post‐4‐HT treatment. The ectopic expression of the Fabp7a‐cerulean fusion protein in the double transgenic fish hearts was confirmed by Western blot analysis (Figure 6B,C). Next, we carried out high‐frequency echocardiography analysis and detected a significantly reduced cardiac function in the *Tg(fabp7a‐OE)*; *Tg(cmlc2‐creER)* double transgenic fish at 3 months post‐4‐HT treatment (Figure 6D). Transmission electron microscopy (TEM) analysis confirmed impaired sarcomere structure and mitochondrial swelling defects (Figure 6E). At the molecular level, aberrant expression of cardiomyopathy markers including *vmhc*, *vmhcl*, and *nppb* were detected, further supporting the cardiomyopathy‐like phenotypes in the *Tg(fabp7a‐OE*); *Tg(cmlc2‐creER)* double transgenic fish (Figure 6F). We next conducted Western blot analysis and detected dysregulated protein homeostasis in the *Tg(fabp7a‐OE)*; *Tg(cmlc2‐creER)* double transgenic fish at three months post‐4‐HT treatment, as indicated by stalled response of LC3II to BafA1 and elevated total ubiquitinated protein level (Figure 7A–C). Additionally, we examined cardiac aging indices and detected elevated staining for p16 and γH2A.X in cardiomyocytes (Figure 7D,E), as well as upregulated mRNA expression of *p21* and *SASP genes* including *il‐1b* and *il‐6* in the *Tg(fabp7a‐OE)*; *Tg(cmlc2‐creER)* double transgenic fish at 3 months post‐4‐HT treatment (Figure 7F). Collectively, these results suggest that cardiomyocyte‐specific overexpression of *fabp7a* caused cardiomyopathy accompanied by dysregulated proteostasis and accelerated cardiac aging phenotypes.

### 2.6 MF6 Treatment Decelerated Cardiac Aging Phenotypes During Normative Aging in the Killifish

**FIGURE 8:**
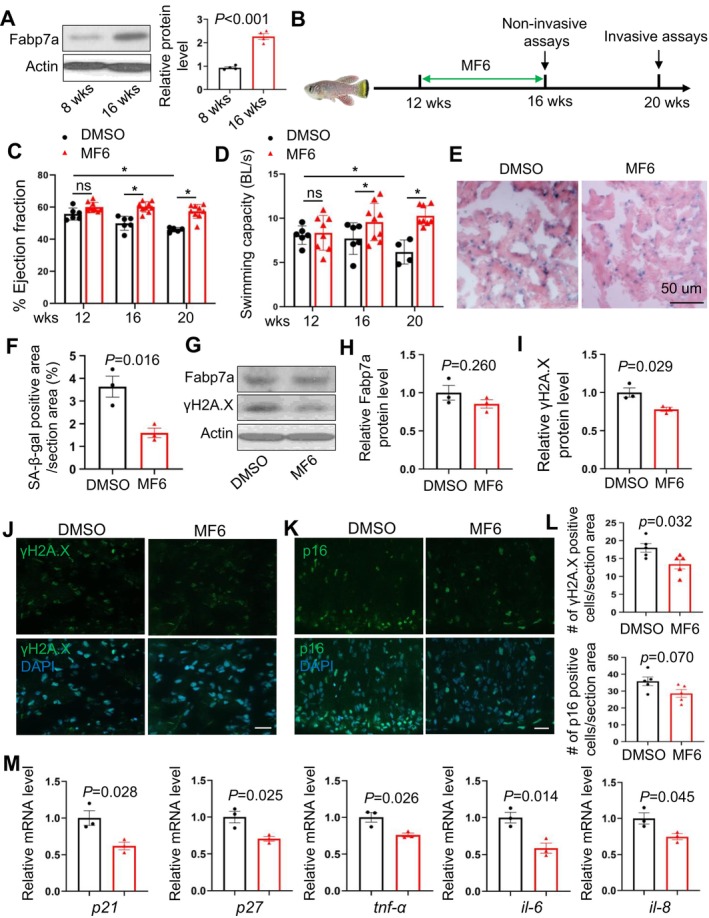
MF6 treatment decelerated cardiac aging indices during normative aging in the turquoise killifish. (A) Representative Western blot and quantification analysis of the Fabp7a protein expression in 8‐week‐old versus 16‐week‐old killifish. *n* = 4, Student *t*‐test. (B) Schematics of the schedules for Fabp7 inhibitor (MF6) treatment from 12‐week‐old to 16‐week‐old killifish. MF6 was delivered once the other day by oral gavage. Non‐invasive assays refer to in vivo phenotyping methods that live fish can tolerate and survive, such as echocardiography and swimming capacity measurement. Invasive assays involve phenotyping methods that require sacrificing the fish to isolate tissues or organs for histological, molecular, and cellular analyses. (C) Ejection fraction (EF, %) quantification in turquoise killifish treated with MF6 (Fabp7a inhibitor, Fabp7‐i) or DMSO control, measured at 12, 16, and 20 weeks of age, showing significantly preserved cardiac function in the MF6‐treated group at 16 and 20 weeks of age. *n* = 5–8, two‐way ANOVA. ns, not significant. *, *p* < 0.05. (D) Swimming capacity assessed as body length per second (BL/s), demonstrating improved physical endurance in MF6‐treated fish at 16 and 20 weeks of age. *n* = 4–8, two‐way ANOVA. ns, not significant. *p < 0.05. (E, F) Representative images (D) and quantification (E) of senescence‐associated β‐galactosidase (SA‐β‐gal) activity staining in the MF6‐treated hearts compared to the DMSO controls at 20 weeks. Scale bar, 50 μm. *n* = 3, Student *t*‐test. (G–I) Representative western blot images (G) and quantification of the Fabp7a (H) and γ‐H2A.X (I) protein levels in the MF6 treated killifish hearts compared to DMSO control. *n* = 3, Student *t*‐test. (J–L) Representative images of γ‐H2A.X (J) and p16 (K) antibody immunofluorescent staining and quantification of γ‐H2A.X‐positive and p16 cell numbers (L) in the MF6‐treated killifish hearts compared to DMSO. Scale bars, 20 μm. *n* = 5, Student *t*‐test. (M) Quantitative RT‐PCR analysis of cellular senescence markers *p21* and *p27* and *SASP* markers including *tnf‐α, il‐6*, and *il‐8* in the MF6 killifish hearts compared to DMSO control. *n* = 3 biological repeats, student *t* test.

To seek direct evidence for functions of Fabp7a in cardiac aging, we utilized the turquoise killifish ( *Nothobranchius furzeri* ), the shortest‐lived vertebrate model organism maintained in captivity (Hu and Brunet 2018), to evaluate the anti‐aging effects of Fabp7a inhibition through pharmacological treatment. We first examined the expression of the Fabp7a protein during normative aging and detected a significant elevation in old fish (Figure 8A). Next, we demonstrated that treatment with FABPs ligand 6 (MF6), a specific inhibitor of Fabp7 (Cheng et al. 2021) via gavage every other day for 4 weeks, significantly restored cardiac function compared to DMSO controls during normative aging (Figure 8B,C). Further assessment of swimming capacity, an indirect measure of cardiac performance, revealed improved physical endurance in MF6‐treated fish as well (Figure 8D). Senescence‐associated (SA) β‐galactosidase activity was significantly reduced following MF6 treatment (Figure 8E,F). At the protein level, while Fabp7a protein expression remains unchanged, the expression levels of γ‐H2A.X, a marker of DNA damage, decreased in the hearts of MF6‐treated killifish (Figure 8G–I). Immunofluorescent staining and quantitative analysis further confirmed a significant reduction in the number of γ‐H2A.X‐positive cells in the hearts of MF6‐treated fish compared to DMSO controls. Additionally, the number of p16‐positive cells, another marker of cellular senescence, was also marginally reduced in the MF6‐treated killifish hearts (Figure 8J–L). At the transcript levels, the expression of cellular senescence markers in turquoise killifish, such as *p21, p27*, and SASP such as *tnf‐α*, *il‐6*, and *il‐8* ^25^ was significantly reduced in MF6‐treated killifish hearts compared to DMSO controls as well (Figure 8M). Collectively, these results suggested that inhibition of Fabp7a through the MF6 treatment significantly mitigated cardiac aging phenotypes during normative aging in the turquoise killifish. Notably, MF6 is also an inhibitor of FABP5, although with an approximately 40‐fold higher Kᴅ (874 vs. 20 nM) than for FABP7 (Cheng et al. 2021). Thus, we cannot exclude the possible off‐target effects of MF6.

## 3 Discussion

### 3.1 Accelerated Cardiac Aging, as Reflected by the Elevated Fabp7a Expression, Is an Important Pathological Event in the *bag3* Cardiomyopathy Model

Prior to this study, it has been well established that dysregulated proteostasis is a primary pathological event in *bag3* cardiomyopathy, significantly contributing to its pathogenesis (Fang et al. 2017; Myers et al. 2018). In this manuscript, we further unveiled that accelerated cardiac senescence also occurs in the *bag3* cardiomyopathy model, as has been reported previously in the anthracycline‐induced cardiomyopathy model (Linders et al. 2024; Seara et al. 2022; Xia et al. 2024). Together, these data prompted future studies to assess whether accelerated cardiac senescence occurs in other types of cardiomyopathies, or if it represents a common pathological feature across cardiomyopathies with diverse etiology. Given that dysregulated proteostasis is one of the 12 hallmarks of normative aging (Lopez‐Otin et al. 2023), it is possible that accelerated cardiac senescence is either correlated with, or even results from dysregulated proteostasis in the heart. More detailed mechanistic studies are warranted to elucidate their relationships between these processes in detail.

Our genetic studies identified upregulated Fabp7a protein expression as an important molecular event in *bag3* cardiomyopathy, which is associated with accelerated cardiac senescence. This finding was further supported by the similarly elevated expression of Fabp7a protein during normative cardiac aging in the turquoise killifish. Phylogenetic analysis indicated that human FABP7 is the closest family member to FABP3, a cardiac‐type FABP, sharing the highest amino acid identity among other FABP members (Smathers and Petersen 2011). Contrary to the previous understanding that FABP7 is primarily a brain‐type FABP which functions in the brain, our data revealed novel cardiac expression and functions of Fabp7a. Transgenic studies demonstrated that cardiomyocyte‐specific overexpression of *fabp7a* is sufficient to drive proteostasis abnormalities and accelerate cardiac senescence. Given that Fabp7 is primarily involved in lipid homeostasis, our data suggest an intrinsic link between lipid metabolism and proteostasis that may play a critical role in the pathogenesis of *bag3* cardiomyopathy. Further experimental studies are necessary to validate this hypothesis and elucidate the underlying mechanisms.

### 3.2 The mTOR‐TFEB‐Fabp7 Signaling Axis Can Be Harnessed for Therapeutic Benefits in *bag3* Cardiomyopathy

We had previously shown that inhibiting mTOR signaling through the *mtor* ^*xu015/+*^ haploinsufficiency mutant exerted cardioprotective effects on *bag3* cardiomyopathy (Ding et al. 2019). In this study, we further reported that cardiomyocyte‐specific overexpression of the zebrafish *tfeb* ortholog, a direct downstream target of the mTOR signaling pathway (Napolitano et al. 2018), ameliorated dilated cardiac structure remodeling, restored the decline in cardiac function and prolonged survival in the *bag3* cardiomyopathy model. These results thus suggest that activation of *tfeb* might confer the cardioprotective effect of mTOR inhibition on *bag3* cardiomyopathy. Consistent with its well‐recognized role in proteostasis (Lu et al. 2021), activation of *tfeb* reduced the accumulation of ubiquitinated protein aggregation in the *bag3* cardiomyopathy hearts and attenuated cellular senescence.

Downstream of the mTOR‐Tfeb axis, *fabp7a* emerged as a candidate therapeutic target gene for *bag3* cardiomyopathy. While expression of *fabp7a* mRNA was downregulated in the hearts of the *mtor* ^*xu015/+*^ haploinsufficiency mutants and *Tg(cmlc2:tfeb)* transgenes, it was aberrantly elevated in the *bag3* cardiomyopathy model. Notably, haploinsufficiency mutation in *fabp7a* largely rescued the cardiomyopathy phenotypes and inhibited accelerated cardiac aging indices in the *bag3* cardiomyopathy model. Conversely, cardiomyocyte‐specific overexpression of *fabp7a* resulted in elevated expression of cellular senescence markers and caused cardiomyopathy. Similar to that in the *bag3* cardiomyopathy model, the expression of Fabp7 protein level was also increased during normative cardiac aging in the killifish. Pharmacological treatment with a Fabp7 specific compound inhibitor decelerated cardiac aging indices in the killifish. Collectively, these genetic and pharmacological studies suggest an mTOR‐Tfeb‐Fabp7a signaling axis involved in cardiac aging which can be harnessed to exert therapeutic benefits in *bag3* cardiomyopathy. To achieve therapeutic benefits, *mtor* and *fabp7a* should be inhibited, while *tfeb* should be activated in the cardiomyocytes. Future studies are warranted to elucidate the mechanisms by which Tfeb negatively regulates Fabp7a expression. Given prior report that TFEB binds to the promoter regions of other human FABP family members, including FABP3, FABP4, and FABP9, to directly regulate their transcriptional expression (Smathers and Petersen 2011), it is reasonable to hypothesize that Tfeb might directly bind to the promoter of Fabp7a to control its transcriptional activity.

### 3.3 Experimentally Testing DEGs Using an F0‐Based Genetic Assay Is an Effective Strategy for Discovering Genetic Modifiers of Cardiomyopathies

The identification of *fabp7a* as a therapeutic modifier gene downstream of the mTOR‐Tfeb signaling axis underscored the power of zebrafish genetics, highlighting an F0‐based genetic assay as a rapid strategy for discovering novel genetic modifiers of an inherited cardiomyopathy. As demonstrated in our recent finding (Ding et al. 2023), it is feasible to achieve high gene knockout efficiency by injecting a single guide RNA designed based on a microhomology‐mediated end joining (MMEJ) sequence. Knockout scores ranging from 76% to 90% for each of the individual candidate genes were achieved in this study. These relatively high knockout scores enable us to test the modifying effects of loss‐of‐function mutations in candidate genes on *bag3* cardiomyopathy within the F0 generation, bypassing the need for lengthy multi‐generational genetic crosses that are typically required for assessing the effects of candidate modifying genes in a disease context. In the era of genomics, where a vast number of candidate genes are often identified through sequencing or transcriptome analysis, this F0‐based genetic screening approach provides a rapid method to identify genetic modifiers. This efficient strategy, proven to be effective here in *bag3* cardiomyopathy, is anticipated to be extendable to other types of cardiomyopathies or any human diseases that can be modeled in zebrafish.

## 4 Methods

### 4.1 Sex as a Biological Variable

Our study examined male and female animals, and similar findings are reported for both sexes.

### 4.2 Animals

Zebrafish *(Danio rerio)* and turquoise killifish *(Nothobranchius furzeri)* were maintained under a 14‐h light–10‐h dark cycle at 28.5°C and handled with care. The animal study protocols were approved by the Mayo Clinic Institutional Animal Care and Use Committee (A3531 for zebrafish, A00003390‐18‐R23 for turquoise killifish). All animal study procedures were performed in accordance with the Guide for the Care and Use of Laboratory Animals published by the US National Institutes of Health (NIH Publication No. 85‐23, revised 1996).

### 4.3 Measurement of the Ventricular Surface Area to Body Weight Ratio

The ventricular surface area (VSA) to body weight (BW) ratio was measured according to a previously published method (Ding et al. 2011). To measure the VSA, the ventricles of individual zebrafish were dissected and imaged next to a millimeter ruler under a Leica MZ FLI III microscope. The largest projection of a ventricle was outlined using ImageJ software. To measure body weight, the fish were anesthetized in 0.16 mg/mL tricaine solution, semi‐dried on a paper towel, and weighed on a scale. The VSA/BW index was then calculated by the largest projection area of the ventricle (in mm^2^) divided by the body weight (in gram) (Zhang et al. 2018).

### 4.4 In Vivo Echocardiography for Adult Zebrafish Hearts

The Vevo 3100 high‐frequency imaging system equipped with a 50 MHz (MX700) linear array transducer (FUJIFILM VisualSonics Inc) was used to measure cardiac function indices in adult zebrafish according to a reported protocol (Zhang et al. 2018). Briefly, acoustic gel (Aquasonic 100, Parker Laboratories Inc) was applied to the surface of the transducer to ensure adequate coupling with the tissue interface. Adult zebrafish at appropriate ages were anesthetized in 0.16 mg/mL tricaine for 3 min and placed ventral side up into a sponge. The MX700 transducer was placed above the zebrafish to provide a sagittal imaging plane of the heart. B‐mode images were acquired with an imaging field view of 9.00 mm in the axial direction and 5.73 mm in the lateral direction, a frame rate of 123 Hz, with medium persistence and a transmit focus at the center of the heart. Image quantification was performed using the VevoLAB workstation. For each index on individual fish, measurements were conducted on 3 independent cardiac cycles to obtain average values.

### 4.5 Transmission Electron Microscopy

For the TEM study, adult zebrafish hearts were dissected and immediately fixed in Trump's solution (4% paraformaldehyde and 1% glutaraldehyde in 0.1 M phosphate buffer [pH, 7.2]) at room temperature (RT) for 1 h, followed by overnight incubation at 4°C. The fixed samples were subsequently processed and imaged at the Mayo Clinic Electron Microscopy Core Facility using a Philips CM10 transmission electron microscope.

### 4.6 SA‐β‐Gal Staining

Senescence‐associated beta‐galactosidase (SA‐β‐gal) staining was performed using the Senescence β‐Galactosidase Staining Kit (Cell Signaling Technology, catalog #: 9860) according to the manufacturer's instructions, with modifications. Briefly, zebrafish and killifish hearts were dissected and immediately fixed overnight at 4°C in 4% paraformaldehyde (PFA) buffered with PBS. The fixed hearts were embedded in a tissue freezing medium and sectioned at 8 μm using a cryostat (Leica CM3050 S). The heart sections were further fixed in the provided fixation solution and stained in the X‐gal staining solution from the kit for 5–12 h at 37°C. X‐gal‐stained sections were imaged using a Zeiss Axioplan 2 microscope (Carl Zeiss). The SA‐β‐gal activity was quantified with Metamorph software.

### 4.7 Quantitative Real‐Time PCR

Total RNA was extracted from an individual adult zebrafish or turquoise killifish ventricle using Trizol reagent (ThermoFisher Scientific) following the manufacturer's instructions. Approximately 100 ng total RNA was used for reverse transcription (RT) and cDNA synthesis using Superscript III First‐Strand Synthesis System (ThermoFisher Scientific). Real‐time quantitative PCR was performed in 96‐well optical plates (ThermoFisher Scientific) using an Applied Biosystem VAii 7 System (ThermoFisher Scientific). Gene expression levels were normalized using the expression levels of glyceraldehyde 3‐phosphate dehydrogenase (*gapdh*) *actin, beta 2 (actb2*) or *18s* by −ΔΔCt (cycle threshold) values.

Primer information for qRT‐PCR in zebrafish is listed as follows:

nppa‐F: 5′‐GATGTACAAGCGCACACGTT‐3′

nppa‐R: 5′‐TCTGATGCCTCTTCTGTTGC‐3′

nppb‐F: 5′‐CATGGGTGTTTTAAAGTTTCTCC‐3′

nppb‐R, 5′‐CTTCAATATTTGCCGCCTTTAC‐3′

vmhc‐F, 5′‐TCAGATGGCAGAGTTTGGAG5‐3′

vmhc‐R, 5′‐GCTTCCTTTACAGTTACAGTCTTTC5‐3′

vmhcl‐F: 5′‐GCGATGCTGAAATGTCTGTT‐3′

vmhcl‐R, 5′‐CAGTCACAGTCTTGCCTCCT‐3′

p21‐F, 5′‐AGGAA AAG CAGCAG AAACG‐3′

p21‐R, 5′‐TGTTG GTCTGT TTG CGCTT‐3′

il‐1b‐F, 5′‐CTGGAGATGTGGACTTCGCA‐3′

il‐1b‐R, 5′‐TCACGCTCTTGGATGACGTT‐3′

il‐6‐F, 5′‐CAGAGACGAGCAGTTTGAGAGA‐3′

il‐6‐R, 5′‐TCAGGACGCTGTAGATTCGC5‐3′

mmp2‐*F*, 5′‐AGCTTTGACGATGACCGCAAATGG‐3′

mmp2*‐R*, 5′‐GCCAATGGCTTGTCTGTTGGTTCT‐3′

β‐actin‐F, 5′‐TTCACCACCACAGCCGAAAGA‐3′

β‐actin‐R, 5′‐TACCGCAA GATTCCATACCCA‐3′.

Primer information for qRT‐PCR in turquoise killifish is listed as follows:

Kp21‐F, 5′‐CGAGCCCTCAGTACTCTAA‐3′

Kp21‐R, 5′‐GAGGTTTGTCGGAGAAGAAG‐3′

Kp27‐F, 5′‐GCTCCTCAATCGCCATATT‐3′

Kp27‐R, 5′‐CATACGTCGAAGTGGAAGAC‐3′

Ktnf‐α‐F, 5′‐CAGGCTCACAAGAGGTTATT‐3′

Ktnf‐α‐R, 5′‐CCAGAGGTCAATCTGTCTTATC‐3′

Kil6‐F, 5′‐GGAGGAATTTCAAGGGAACATA‐3′

Kil6‐R, 5′‐CCTCAGGAGAACCATGTAGA‐3′

Kil8‐F, 5′‐ACAAATCCTGACCACAAGTAG‐3′

Kil8‐R, 5′‐ATCGTATTCACCATCATGTCTC‐3′

K18s‐F, 5′‐CCGACATTGACCTCAACAA‐3′

K18s‐R, 5′‐TGGCTGTATTTGCCATCC‐3′

### 4.8 Antibody Immunostaining

Heart samples dissected from adult zebrafish were embedded in a tissue freezing medium and sectioned at 8 μm using a cryostat (Leica CM3050 S). Sections were air dried for 30 min (mins) at room temperature and fixed with 4% PBS‐buffered paraformaldehyde (PFA) for 7 min, permeabilized with 0.1% Triton X‐100 in PBD (1X PBS, 1% BSA, 1% DMSO) for 45 min, blocked with 2% sheep serum/PBD for 25 min, and incubated with primary antibodies overnight at 4°C. Primary antibody‐stained sections were then washed in PBD three times and incubated with secondary antibodies (Alexa Fluor anti‐Rabbit 488, ThermoFisher Scientific, catalog #A11008; Alexa Fluor anti‐Mouse 568, ThermoFisher Scientific, catalog #A11001) at RT for 1 h, washed with PBD three times, and transferred to a slide with a mounting medium containing DAPI (Vector, H‐1200). Stained sections were imaged with a Zeiss Axioplan 2 microscope equipped with ApoTome and AxioVision software (Carl Zeiss). The following primary antibodies were used: anti‐MEF2(A + C) (1:300, Abcam, catalog #197070), anti‐p16 (1:100, Santa Cruz, catalog #sc‐1661), anti‐γH2A.X (phosphor Ser139) (1:200, GeneTex, catalog #GTX127342), anti‐α‐actinin (1:300, Sigma, catalog #A7811), anti‐Fabp7 (1:100, GeneTex, catalog #GTX121467).

### 4.9 Western Blotting

Either pooled zebrafish embryos at 3 days post‐fertilization or individually dissected adult zebrafish heart ventricles were transferred to the RIPA lysis buffer (Sigma‐Aldrich) supplemented with the complete protease inhibitor cocktail (MilliporeSigma) and homogenized using a Bullet Blender tissue homogenizer (Next Advance Inc). For analysis of aggregated ubiquitinated protein levels, insoluble protein aggregate fractions were prepared as previously described, with modification (Zhu et al. 2017). Briefly, individual adult fish hearts were homogenized in 20 μL lysis buffer (100 mM HEPES, 1% Triton X100, 300 mM NaCl) supplemented with 1 mM phenylmethylsulphonyl fluoride and 1 × complete protease inhibitor cocktail (MilliporeSigma), pre‐cleared at 2000 g for 2 min, and then centrifuged at 12,000*g* for 10 min to separate extracts into soluble and insoluble fractions. The isolated insoluble fraction was further lysed by adding an additional 0.5% SDS. The resultant protein lysates were subjected to western blotting using a standard protocol. The following primary antibodies were used: anti‐γH2A.X (phosphor Ser139) (1:1000, GeneTex, catalog #GTX127342), anti‐LC3 (1:2000, Cell Signaling Technology, catalog #12741), anti‐β‐actin (1:8000, Santa Cruz Biotechnology Inc., catalog #sc‐1615), anti‐Ubiquitin (1:1000, ThermoFisher Scientific, catalog #PA5‐17067; or 1:1000, Cell Signaling Technology, catalog #3936, or 1:11000, Santa Cruz Biotechnology Inc., catalog #sc‐8017), anti‐Fabp7 (1:1000, GeneTex, catalog #GTX121467), anti‐Gapdh (1:4000, Santa Cruz Biotechnology Inc., catalog #sc), anti‐HSP70 (1:1000, Santa Cruz Biotechnology Inc., catalog #sc‐66049), and anti‐HSC70 (1:1000, Santa Cruz Biotechnology Inc., catalog #sc‐7298).

### 4.10 RNA‐Seq Analysis

RNA‐seq data acquisition and analysis were performed according to a previously published protocol (Ding et al. 2019; Zhang et al. 2018). Briefly, total RNA was extracted from dissected ventricular tissue of 6‐month‐old *mtor* ^*xu015/+*^ mutant, *Tg(cmlc2:tfeb)* transgenic fish and corresponding WT siblings. Five ventricles were pooled as one sample and three biological replicates for each genotype were sequenced using the HiSeq 2000 platform (Illumina) with a 50‐bp paired‐end sequencing protocol in the Mayo Clinic DNA Sequencing Core Facility. Raw RNA‐seq reads for each sample were originally aligned with TopHat (Version 2.1.1) to the zebrafish genome assembly (Zv9) using the Ensembl annotation Zv9 (Danio_rerio.Zv9.79.gtf) and later with the updated genome assembly GRCz11 (Genome assembly GRCz11). Gene expression was quantified using Cufflinks (Version 2.2.1). Differential gene expression across different groups was determined based on fold change and a false discovery rate of less than 0.05 according to the Cuffdiff script from Cufflinks. Unsupervised hierarchical clustering was performed using Pearson correlation and scaled based on the fragments per kilobase of transcript per million mapped reads value with the pheatmap R package (https://github.com/raivokolde/pheatmap). Both the primary RNA‐seq raw and processed datasets have been deposited in GEO under the accession number GSE269725.

### 4.11 MMEJ‐Based Single Guide RNA Design and F0 Based Genetic Testing

MMEJ‐based single guide RNA design and F0 injection were performed according to a recently published protocol (Ding et al. 2023). Briefly, targeted exon sequences of genes were uploaded to an online algorithm, MENTHU (http://genesculpt.org/menthu/). Target guide RNA sequences with high scores were selected from predicted MMEJ loci. Single guide RNAs (sgRNAs) with appropriate chemical modifications were synthesized and obtained from Synthego (Synthego Corporation). sgRNAs were dissolved in nuclease‐free duplex buffer (Integrated DNA Technologies, 11‐01‐03‐01) and diluted to 5 μM as working concentrations. The sgRNA‐Cas9 protein (sgRNP) complex was then assembled and injected into one‐cell staged zebrafish embryos to obtain MMEJ‐injected F0 embryos. Individual MMEJ sgRNP‐injected embryos were harvested and subjected to knockdown efficiency measurement and calculation. Sequences were obtained via Sanger sequencing at Genewiz (https://clims4.genewiz.com/CustomerHome/Index), and the knockdown scores were obtained using the R code‐based open access software Inference of CRISPR Edits (ICE) v2 CRISPR Analysis Tool (https://www.synthego.com/products/bioinformatics/crispr‐analysis) (Brinkman and van Steensel 2019). Primer information for assessing knockdown efficiency is listed as follows: bscl2‐MJ‐F1, 5′‐TGAACAGAAATGGAGCGTG‐3′, bscl2‐MJ‐R1, AATCAAGAATGGTCACCTGATG‐3′; fabp7a‐MJ‐F1, 5′‐GCATGTGTAAGGTGCAGTAG‐3′, fabp7a‐MJ‐R1, 5′‐GTGGTTTCATCAAACTCCTCTC‐3′; cidec‐MJ‐F1, 5′‐ATGTGCGTTGTGTGTCAC‐3′, cidec‐MJ‐R1, 5′‐TGTTTGCCGGGTTCTTTC‐3′; edn1‐MJ‐F1, AGCTGTCATTGCATGACTTG‐3′, edn1‐MJ‐R1, 5′‐CACTGTCTCTGTGGTTTGTC‐3′.

### 4.12 Generation of Cardiomyocyte‐Specific Overexpression of *fabp7a* Transgenic Line

The *Tg(βactin2:loxP‐mCherry‐stop‐loxP‐fabp7a‐cerulean)* transgenic line was generated using the Tol2/Gateway system according to a previously published approach (Wang et al. 2021). The zebrafish full length *fabp7a* cDNA was RT‐PCR amplified using a forward primer 5′‐ATCGGAATTCTGGCCACCATGGTCGATGCATTTTGTGCCACTTGGG‐3′ and a reverse primer 5′‐ATCGGCGGCCGCTGCCTTCTCGTATGTGCGCACGGCCTG‐3′ introduced with EcoRI and NotI cut sites. The resultant PCR fragment was digested with EcoRI and NotI and inserted into the pENTRI1‐loxP‐mCherry‐stop‐loxP vector to generate pENTRI‐loxP‐mCherry‐stop‐loxP‐fabp7a, which was then recombined with p5E‐βactin2, p3E‐cerulean‐polyA, and pDestTol2pA to obtain the final construct: Tg(βactin2: loxP‐mCherry‐stop‐loxP‐fabp7a‐cerulean) using Gateway LR Clonase II Plus Enzyme (ThermoFisher). Founder zebrafish (F0) were identified based on positive mCherry fluorescence and genotyping PCR. F1 stable transgenic fish were obtained by outcrossing. The resultant *Tg(βactin2: loxP‐mCherry‐stop‐loxP‐fabp7a‐cerulean)* fish were then further outcrossed with the *Tg(cmlc2:creERT2)* fish to generate double transgenic fish, which were then incubated with 1 μM 4‐HT in system water for 24 h to induce loxP‐Cre recombination.

### 4.13 Proteasome Activity Assay

Hearts were dissected from designated fish at 6 months of age, and each individual heart was collected in a 1.5 mL Eppendorf tube. Samples were homogenized with a Bullet Blender tissue homogenizer (Next Advance Inc) in 20 μL of Tris buffer (50 mM Tris, pH 7.5, 1 mM EDTA). The resulting tissue lysates were centrifuged at 17,000*g* for 10 min at 4°C, and the supernatant was transferred to a new Eppendorf tube for protein quantification and proteasome activity assay, following the manufacturer's instructions (UBPBio, Proteasome Activity Fluorometric Assay Kit, catalog # J4110). Briefly, 1 μg of total protein was diluted in 50 μL of 1× Proteasome Assay Buffer and mixed with 50 μL of substrate containing 50 μM of Suc‐LLVY‐AMC in a 96‐well plate. The mixture was then incubated at 37°C for 10 min, and dynamic fluorescence was measured using a microplate reader (FLUOstar Omega from BMG LABTECH) at excitation/emission wavelengths of 355/460 nM once every hour for 3 h.

### 4.14 Statistics

The unpaired two‐tailed Student's *t*‐test was used to compare two groups. One‐way analysis of variance (ANOVA) was used to assess differences among three or more groups. For the animal survival rate comparison, the log‐rank test was used to determine the difference. For all dot plots, each value represents the mean ± standard error (SE). All statistical analyses were performed with GraphPad Prism 7. For the post hoc analysis, Tukey's test was employed to confirm the findings.

## Author Contributions

**Yonghe Ding**, **Xueying Lin**, and **Xiaolei Xu:** conceptualization. **Yonghe Ding**, **Xueling Ma**, **Feixiang Yan:** methodology. **Yonghe Ding**, **Xueling Ma**, **Feixiang Yan**, and **Yuji Zhang:** software. **Yonghe Ding** and **Xiaolei Xu:** validation. **Yonghe Ding**, **Xueling Ma**, **Feixiang Yan**, **Yuji Zhang**, and **Xiaolei Xu:** formal analysis. **Yonghe Ding** and **Xiaolei Xu:** investigation. **Yonghe Ding**, **Xueling Ma**, **Feixiang Yan**, **Baul Yoon**, and **Wei Wei** **:** writing – original draft. **Yonghe Ding** and **Xiaolei Xu:** writing – review and editing. **Yonghe Ding**, **Baul Yoon**, and **Xiaolei Xu:** data curation. **Xueying Lin** and **Xiaolei Xu:** supervision. **Xiaolei Xu:** project administration. **Xiaolei Xu:** funding acquisition.

## Conflicts of Interest

The authors declare no conflicts of interest.

## Supporting information


**Data S1:** acel70216‐sup‐0001‐DataS1.pptx.

## Data Availability

All the data that support the findings of this study are available upon reasonable request.
