# Supplementary material for: An mTOR‐Tfeb‐Fabp7a Axis Ameliorates bag3 Cardiomyopathy via Decelerating Cardiac Aging
Source: Aging Cell. 2025 Sep 8;24(11):e70216. doi: 10.1111/acel.70216 (PMC12611321; doi:10.1111/acel.70216)
Supplement: Supplementary file 1 — Data S1: acel70216‐sup‐0001‐DataS1.pptx. [file ACEL-24-e70216-s001.pptx]

## Slide 1
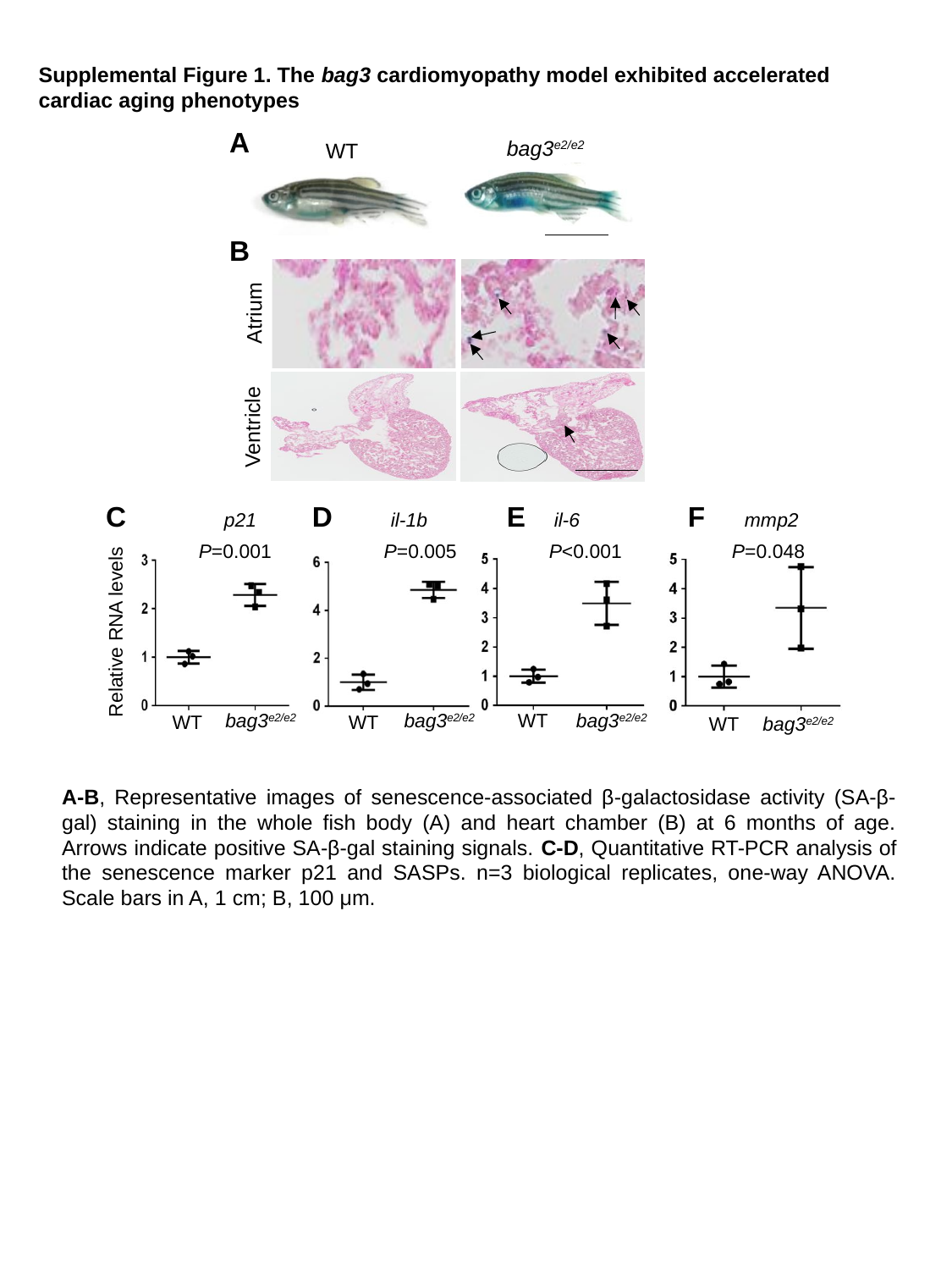

Supplemental Figure 1. The bag3 cardiomyopathy model exhibited accelerated cardiac aging phenotypes
A
bag3e2/e2
WT
B
Atrium
Ventricle
C
D
E
F
p21
il-1b
il-6
mmp2
P=0.001
P=0.005
P<0.001
P=0.048
Relative RNA levels
bag3e2/e2
bag3e2/e2
WT
bag3e2/e2
WT
WT
WT
bag3e2/e2
A-B, Representative images of senescence-associated β-galactosidase activity (SA-β-gal) staining in the whole fish body (A) and heart chamber (B) at 6 months of age. Arrows indicate positive SA-β-gal staining signals. C-D, Quantitative RT-PCR analysis of the senescence marker p21 and SASPs. n=3 biological replicates, one-way ANOVA. Scale bars in A, 1 cm; B, 100 μm.

## Slide 2
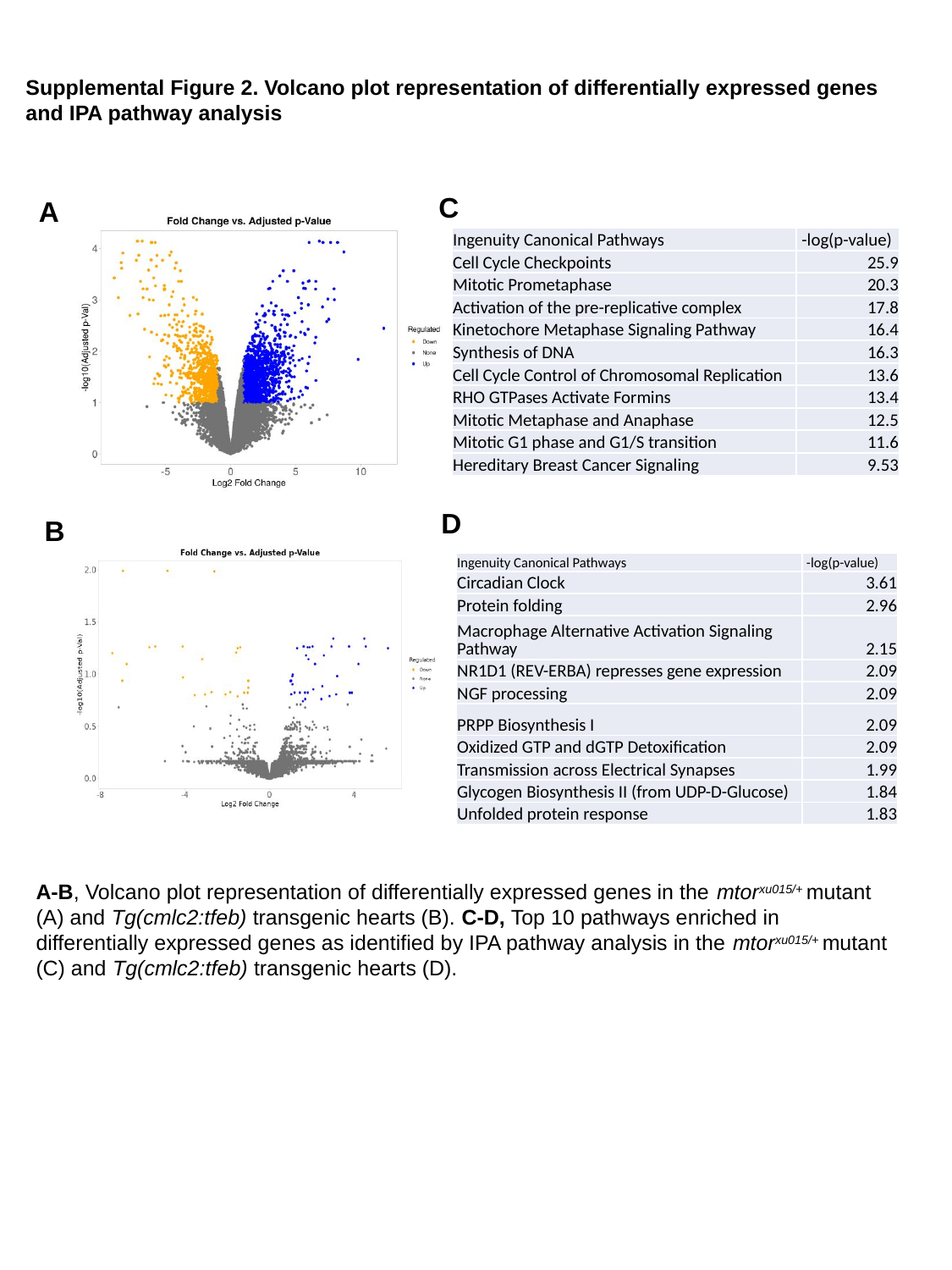

Supplemental Figure 2. Volcano plot representation of differentially expressed genes and IPA pathway analysis
C
A
| Ingenuity Canonical Pathways | -log(p-value) |
| --- | --- |
| Cell Cycle Checkpoints | 25.9 |
| Mitotic Prometaphase | 20.3 |
| Activation of the pre-replicative complex | 17.8 |
| Kinetochore Metaphase Signaling Pathway | 16.4 |
| Synthesis of DNA | 16.3 |
| Cell Cycle Control of Chromosomal Replication | 13.6 |
| RHO GTPases Activate Formins | 13.4 |
| Mitotic Metaphase and Anaphase | 12.5 |
| Mitotic G1 phase and G1/S transition | 11.6 |
| Hereditary Breast Cancer Signaling | 9.53 |
D
B
| Ingenuity Canonical Pathways | -log(p-value) |
| --- | --- |
| Circadian Clock | 3.61 |
| Protein folding | 2.96 |
| Macrophage Alternative Activation Signaling Pathway | 2.15 |
| NR1D1 (REV-ERBA) represses gene expression | 2.09 |
| NGF processing | 2.09 |
| PRPP Biosynthesis I | 2.09 |
| Oxidized GTP and dGTP Detoxification | 2.09 |
| Transmission across Electrical Synapses | 1.99 |
| Glycogen Biosynthesis II (from UDP-D-Glucose) | 1.84 |
| Unfolded protein response | 1.83 |
A-B, Volcano plot representation of differentially expressed genes in the mtorxu015/+ mutant (A) and Tg(cmlc2:tfeb) transgenic hearts (B). C-D, Top 10 pathways enriched in differentially expressed genes as identified by IPA pathway analysis in the mtorxu015/+ mutant (C) and Tg(cmlc2:tfeb) transgenic hearts (D).

## Slide 3
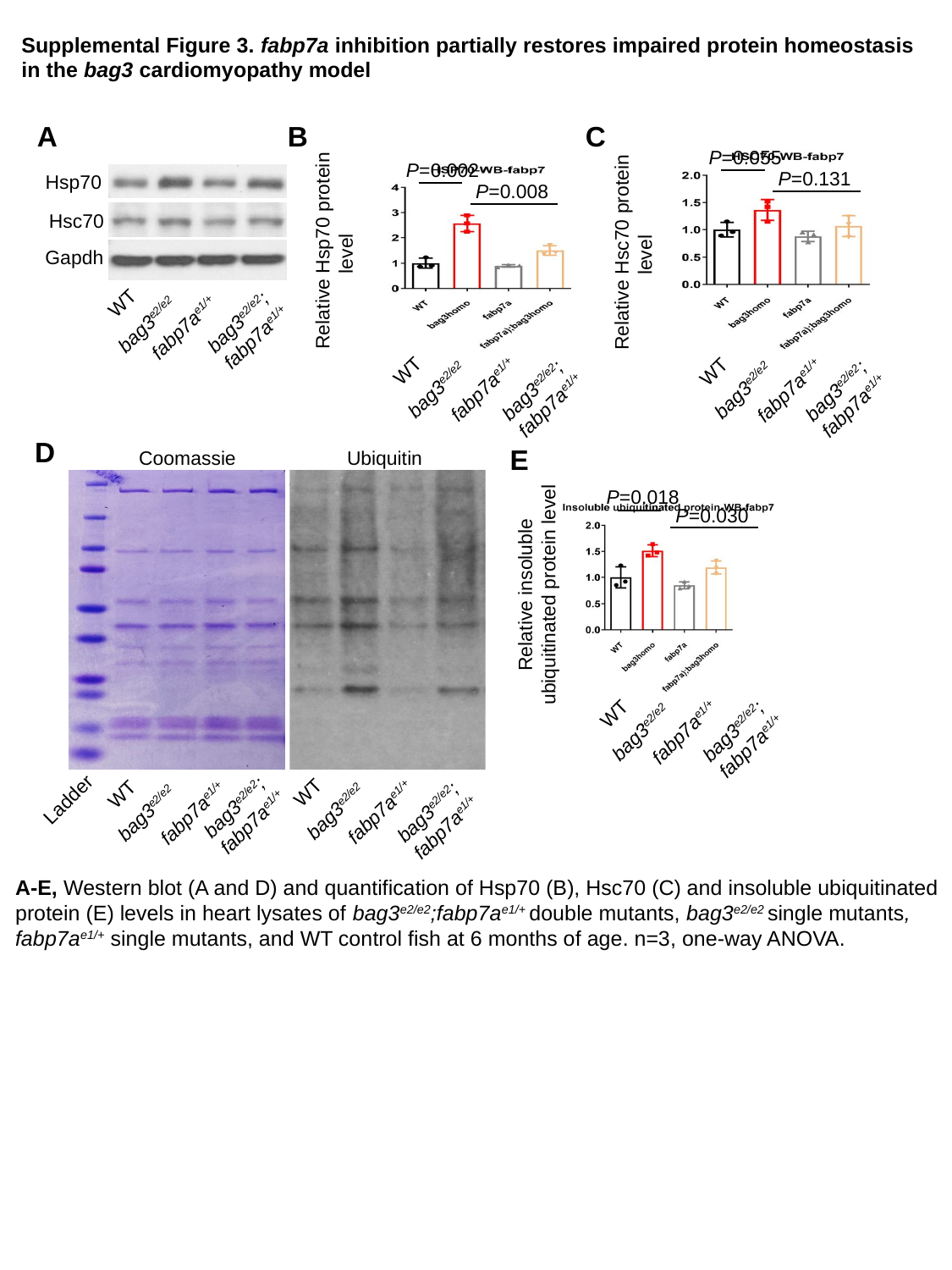

Supplemental Figure 3. fabp7a inhibition partially restores impaired protein homeostasis in the bag3 cardiomyopathy model
A
B
C
P=0.055
P=0.002
P=0.131
Hsp70
P=0.008
Hsc70
Relative Hsp70 protein level
Relative Hsc70 protein level
Gapdh
WT
bag3e2/e2;
fabp7ae1/+
bag3e2/e2
 fabp7ae1/+
WT
WT
bag3e2/e2;
fabp7ae1/+
bag3e2/e2;
fabp7ae1/+
bag3e2/e2
 fabp7ae1/+
bag3e2/e2
 fabp7ae1/+
D
E
Coomassie
Ubiquitin
P=0.018
P=0.030
Relative insoluble ubiquitinated protein level
WT
bag3e2/e2;
fabp7ae1/+
bag3e2/e2
 fabp7ae1/+
WT
WT
bag3e2/e2;
fabp7ae1/+
Ladder
bag3e2/e2;
fabp7ae1/+
bag3e2/e2
bag3e2/e2
 fabp7ae1/+
 fabp7ae1/+
A-E, Western blot (A and D) and quantification of Hsp70 (B), Hsc70 (C) and insoluble ubiquitinated protein (E) levels in heart lysates of bag3e2/e2;fabp7ae1/+ double mutants, bag3e2/e2 single mutants, fabp7ae1/+ single mutants, and WT control fish at 6 months of age. n=3, one-way ANOVA.

## Slide 4
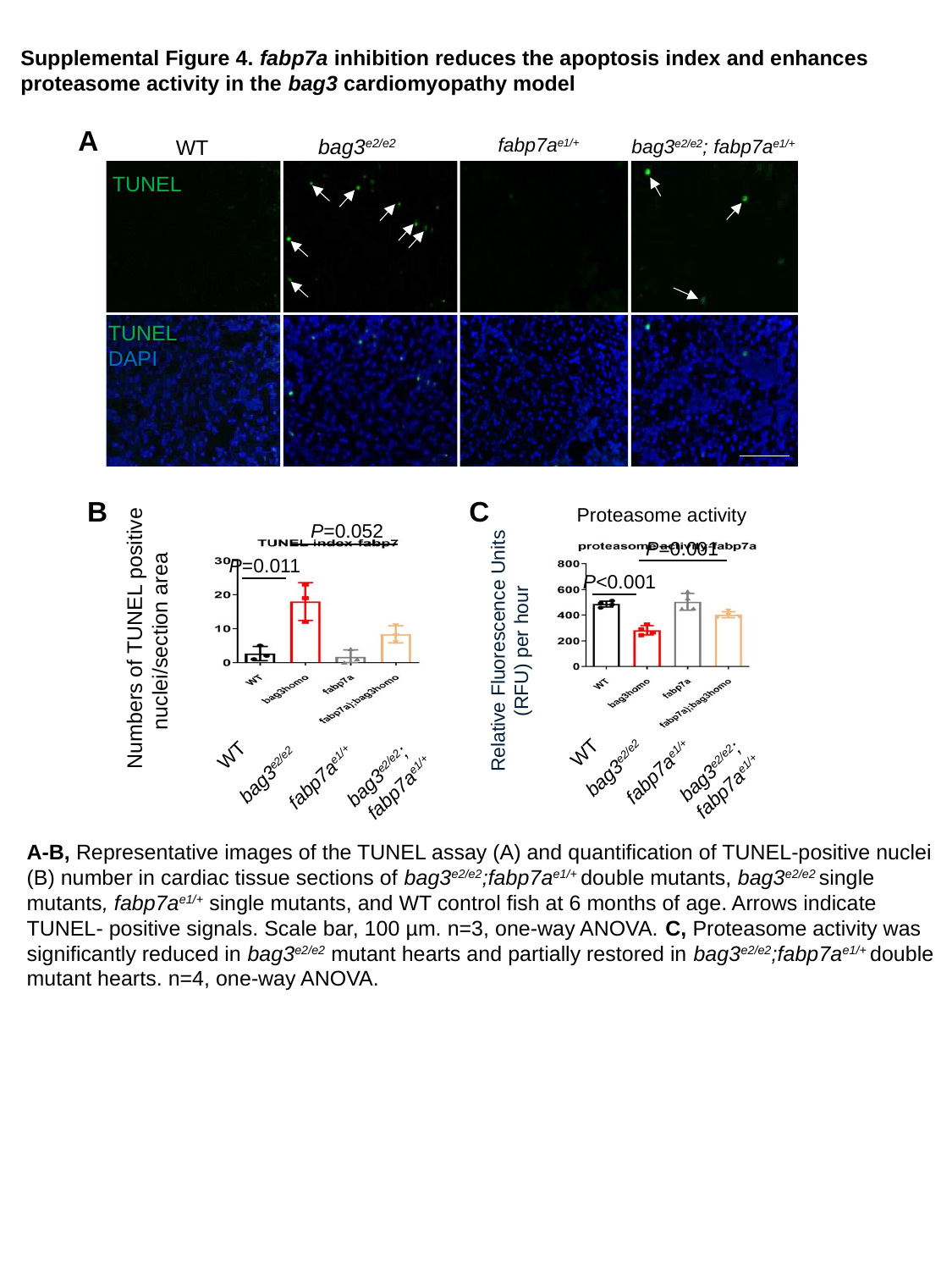

Supplemental Figure 4. fabp7a inhibition reduces the apoptosis index and enhances proteasome activity in the bag3 cardiomyopathy model
A
fabp7ae1/+
bag3e2/e2
bag3e2/e2; fabp7ae1/+
WT
TUNEL
TUNEL
DAPI
B
C
Proteasome activity
P=0.052
P=0.001
P=0.011
P<0.001
Numbers of TUNEL positive nuclei/section area
Relative Fluorescence Units
(RFU) per hour
WT
WT
bag3e2/e2;
fabp7ae1/+
bag3e2/e2
bag3e2/e2
bag3e2/e2;
 fabp7ae1/+
 fabp7ae1/+
 fabp7ae1/+
A-B, Representative images of the TUNEL assay (A) and quantification of TUNEL-positive nuclei (B) number in cardiac tissue sections of bag3e2/e2;fabp7ae1/+ double mutants, bag3e2/e2 single mutants, fabp7ae1/+ single mutants, and WT control fish at 6 months of age. Arrows indicate TUNEL- positive signals. Scale bar, 100 µm. n=3, one-way ANOVA. C, Proteasome activity was significantly reduced in bag3e2/e2 mutant hearts and partially restored in bag3e2/e2;fabp7ae1/+ double mutant hearts. n=4, one-way ANOVA.

## Slide 5
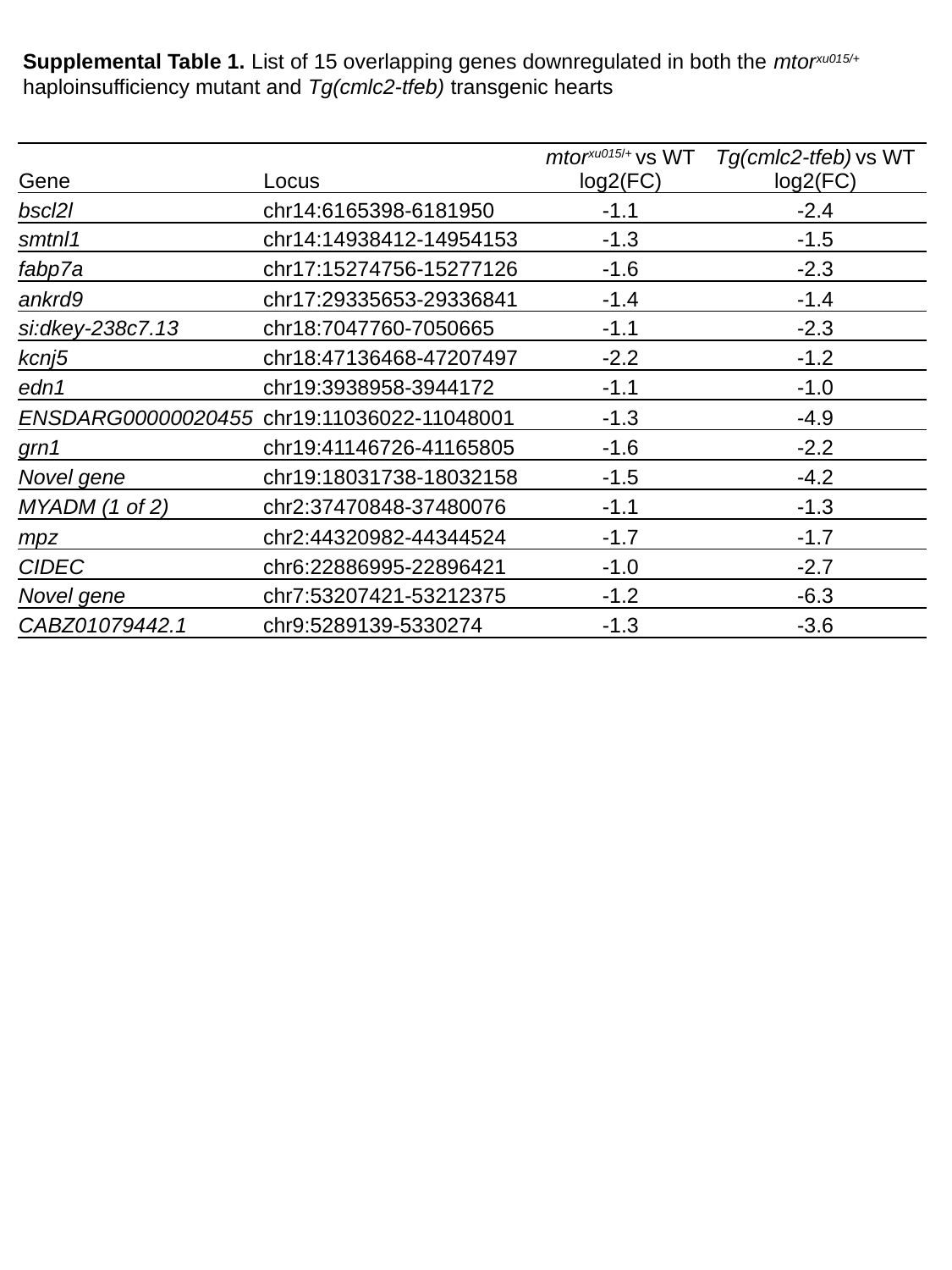

Supplemental Table 1. List of 15 overlapping genes downregulated in both the mtorxu015/+ haploinsufficiency mutant and Tg(cmlc2-tfeb) transgenic hearts
| Gene | Locus | mtorxu015/+ vs WT log2(FC) | Tg(cmlc2-tfeb) vs WT log2(FC) |
| --- | --- | --- | --- |
| bscl2l | chr14:6165398-6181950 | -1.1 | -2.4 |
| smtnl1 | chr14:14938412-14954153 | -1.3 | -1.5 |
| fabp7a | chr17:15274756-15277126 | -1.6 | -2.3 |
| ankrd9 | chr17:29335653-29336841 | -1.4 | -1.4 |
| si:dkey-238c7.13 | chr18:7047760-7050665 | -1.1 | -2.3 |
| kcnj5 | chr18:47136468-47207497 | -2.2 | -1.2 |
| edn1 | chr19:3938958-3944172 | -1.1 | -1.0 |
| ENSDARG00000020455 | chr19:11036022-11048001 | -1.3 | -4.9 |
| grn1 | chr19:41146726-41165805 | -1.6 | -2.2 |
| Novel gene | chr19:18031738-18032158 | -1.5 | -4.2 |
| MYADM (1 of 2) | chr2:37470848-37480076 | -1.1 | -1.3 |
| mpz | chr2:44320982-44344524 | -1.7 | -1.7 |
| CIDEC | chr6:22886995-22896421 | -1.0 | -2.7 |
| Novel gene | chr7:53207421-53212375 | -1.2 | -6.3 |
| CABZ01079442.1 | chr9:5289139-5330274 | -1.3 | -3.6 |
